# TRPC1 Deletion Causes Striatal Neuronal Cell Apoptosis and Proteomic Alterations in Mice

**DOI:** 10.3389/fnagi.2018.00072

**Published:** 2018-03-20

**Authors:** Dian Wang, Haitao Yu, Benhong Xu, Hua Xu, Zaijun Zhang, Xiaohu Ren, Jianhui Yuan, Jianjun Liu, Yi Guo, Peter S. Spencer, Xifei Yang

**Affiliations:** ^1^College of Pharmacy, Jinan University, Guangzhou, China; ^2^Key Laboratory of Modern Toxicology of Shenzhen, Shenzhen Center for Disease Control and Prevention, Shenzhen, China; ^3^Institute of New Drug Research and Guangzhou, Key Laboratory of Innovative Chemical Drug Research in Cardio-Cerebrovascular Diseases, Jinan University College of Pharmacy, Guangzhou, China; ^4^Department of Neurology, Second Clinical College, Jinan University, Shenzhen, China; ^5^Department of Neurology, School of Medicine, Oregon Institute of Occupational Health Sciences, Oregon Health and Science University, Portland, OR, United States

**Keywords:** TRPC1 deletion, apoptosis, striatum, proteomics, ER stress response

## Abstract

Transient receptor potential channel 1 (TRPC1) is widely expressed throughout the nervous system, while its biological role remains unclear. In this study, we showed that TRPC1 deletion caused striatal neuronal loss and significantly increased TUNEL-positive and 8-hydroxy-2′-deoxyguanosine (8-OHdG) staining in the striatum. Proteomic analysis by two-dimensional fluorescence difference gel electrophoresis (2D-DIGE) coupled with mass spectrometry (MS) revealed a total of 51 differentially expressed proteins (26 increased and 25 decreased) in the stratum of TRPC1 knockout (TRPC1^−/−^) mice compared to that of wild type (WT) mice. Bioinformatics analysis showed these dysregulated proteins included: oxidative stress-related proteins, synaptic proteins, endoplasmic reticulum (ER) stress-related proteins and apoptosis-related proteins. STRING analysis showed these differential proteins have a well-established interaction network. Based on the proteomic data, we revealed by Western-blot analysis that TRPC1 deletion caused ER stress as evidenced by the dysregulation of GRP78 and PERK activation-related signaling pathway, and elevated oxidative stress as suggested by increased 8-OHdG staining, increased NADH dehydrogenase (ubiquinone) flavoprotein 2 (NDUV2) and decreased protein deglycase (DJ-1), two oxidative stress-related proteins. In addition, we also demonstrated that TRPC1 deletion led to significantly increased apoptosis in striatum with concurrent decrease in both 14–3–3Z and dynamin-1 (D2 dopamine (DA) receptor binding), two apoptosis-related proteins. Taken together, we concluded that TRPC1 deletion might cause striatal neuronal apoptosis by disturbing multiple biological processes (i.e., ER stress, oxidative stress and apoptosis-related signaling). These data suggest that TRPC1 may be a key player in the regulation of striatal cellular survival and death.

## Introduction

Transient receptor potential (TRP) channels function as cation-conducting protein sensors that respond to physical (temperature, pressure, pH, voltage) and chemical (proteins, lipids, metals) inputs, and thereby trigger signal transduction events. They are composed of six subfamilies: the TRPC (canonical), TRPV (vanilloid), TRPM (melastatin), TRPP (polycystin), TRPML (mucolipin) and TRPA (ankyrin; Gees et al., 2010). Most of the known functions of TRP proteins are conserved from protists, worms, and flies to humans (Montell, 2005; Damann et al., 2008). TRP proteins play important roles in the regulation of calcium signaling and are activated not only by G protein-coupled receptors (GPCRs) and receptor tyrosine kinases (RTKs) but also by chemo-, thermo- and mechanical stimulation (Venkatachalam and Montell, 2007). Dysregulated TRP proteins are connected with a large number of disorders, including neurodegenerative diseases. Although functions of a variety of TRP proteins have been studied extensively, understanding of how TRP proteins function in specific tissues, including brain tissue, is still limited.

TRP channels are widely expressed in the central nervous system (CNS) and the TRPC protein profiles change during embryonic CNS development (Strübing et al., 2003; Zeng et al., 2016). TRPCs play pivotal roles in the control of cell proliferation and differentiation through regulation of gene expression and membrane dynamics (Zeng et al., 2016). Among the TRPC proteins, transient receptor potential channel 1 (TRPC1) is one of the most extensively studied TRPC channels in CNS tissue. TRPC1 protected neuronal cells against neurotoxins and apoptosis of dopaminergic SH-SY5Y cells induced by 1-methyl-4-phenylpyridinium ion (MPP^+^; Selvaraj et al., 2009; Arshad et al., 2014; Wang et al., 2016). In addition, TRPC1 knockout mice (TRPC1^−/−^) exhibited loss of dopaminergic neurons in substantia nigra, which occurs in the pathogenesis of neurodegenerative diseases (Selvaraj et al., 2012). The striatum, a critical part of the motor and reward systems, showed neuronal damage under decreased TRPC1 expression (Hong et al., 2015). Previously, we demonstrated that the deletion of mouse TRPC1 caused spatial memory impairment and dysregulation of protein expression in the hippocampus (Xing et al., 2016). However, the potential effect on neuronal survival in specific brain regions such as the striatum, and the underlying mechanisms remain to be elucidated.

Consistent with previous reports (Selvaraj et al., 2009, 2012), we found significant neuronal loss and apoptosis in the striatum of TRPC1^−/−^ vs. wild type (WT) mice. We use immunofluorescent staining, two-dimensional fluorescence difference gel electrophoresis (2D-DIGE), coupled with mass spectrometry (MS) and bioinformatics analysis, to investigate the change of neuronal survival/apoptosis in striatum of TRPC1^−/−^ mice to illuminate molecular mechanisms underlying the role of TRPC1.

## Materials and Methods

### Animals

The TRPC1^−/−^ mice were obtained from Prof. Lutz Birnbaumer (NIEHS, US) and the WT mice were purchased from Vital River Laboratory Animal Technology Co. Ltd (Beijing, China). A total of 22 mice (8 months old) were used in this study. Animals had free access to food and water, and were maintained in a room with stable humidity (50%–60%) and controlled temperature (23–25°C) on a 12-h light/12-h dark cycle.

Animal treatment and care were performed in accordance with the Principles of Laboratory Animal Care (NIH publication No. 85-23, revised in 1985) and the Regulations for Animal Care and Use from the Committee of the Experimental Animal Center at Shenzhen Center for Disease Control and Prevention in Shenzhen, Guangdong Province, China. This animal study was approved by Shenzhen Center for Disease Control and Prevention Ethics Committee. Efforts were made to minimize animal suffering and reduce the number of mice used for experiments.

### Sample Preparation

Mice were anesthetized with 4% chloral hydrate, the thorax and heart exposed, and the animal perfused via the left ventricle with 0.9% NaCl to remove circulating blood. The brain was excised, fixed by immersion in 4% (w/v) paraformaldehyde fixative in 0.1 M phosphate buffer solution (pH = 7.4). Afterwards, the complete rat brain was removed and stored in 4% (w/v) paraformaldehyde fixative for 24 h at 4°C, then dehydrated and embedded in paraffin. Coronal sections 5 μm thick from areas of interest were prepared for analysis.

### TUNEL Assay

For the measurement of apoptosis, the TUNEL assay was measured by the DeadEnd^TM^ Fluorometric TUNEL System (Promega, Fitchburg, WI, USA) as described in the instruction manual. Briefly, brain sections were deparaffinized, and stepwise hydrated in decreasing concentrations of ethanol (100%, 95%, 85%, 70%, and 50%), each for 3 min. The tissue was then immersed in 0.85% NaCl for 5 min, washed with 1× PBS for 5 min, fixed in 4% paraformaldehyde for 15 min, and washed twice with PBS for 5 min each time. One hundred microliter of 20 μg/ml proteinase K solution was added into each sample and incubated for 8 min at RT. Then the tissues were equilibrated for 5 min, and 50 μl of TdT reaction mix solution added and tissues incubated for 60 min at 37°C in a humidified chamber in the dark. The reaction was stopped by immersing the tissues in 2× saline sodium citrate (SSC) (saline sodium citrate) for 15 min. Finally, the tissues were stained with 4′,6-diamidino-2-phenylindole (DAPI) for 3 min, and examined with a light microscope (Olympus 1× 51, Tokyo, Japan).

### Immunofluorescent Staining

For immunofluorescence, 5 μm-thick coronal cryostat sections were rinsed in PBS after dewaxing and rehydration, and then immersed in 0.01 M citrate buffer (pH 6.0) at 95–100°C for 5 min. The treated sections were blocked for 60 min in blocking buffer solution (0.1% Triton^TM^ X-100/1× PBS 5% normal serum). Anti-8-hydroxyguanosine (goat polyclonal, diluted 1:200, Abcam, Cambridge, UK, ab10802) and anti- NeuN (rabbit monoclonal, diluted 1:300, Abcam, ab177487) were used and processed at 4°C overnight. After washing with PBST, all secondary antibodies were incubated for 1 h at RT in the dark. ThermoFisher Scientific (Waltham, MA, USA) Alexa Fluor 488 donkey anti-goat (Invitrogen) and Alexa Fluor 488 goat anti-rabbit (Invitrogen) were used for the recognition of primary antibodies, respectively. After incubation, the sections were washed with PBST and then stained with DAPI (Beyotime Institute of Biotechnology, Haimen, China) for 5 min to reveal the nuclei. The images were observed with a laser scanning confocal microscope (Leica, Wetzlar, Germany).

### Protein Sample Preparation for 2D DIGE

The brain samples were suspended in DIGE-specific lysis buffer (7 M urea, 2 M thiourea, 30 mM Tris-HCl, 4% CHAPS, pH 8.5), ultrasonicated for 3 min in cycles of 4 s on and 6 s off at 45% power via a Fisher 550 Sonic Dismembrator (Pittsburgh, PA, USA), and then placed on ice for 30 min. The mixture was centrifuged subsequently at 20,000 *g* for 60 min at 4°C, followed by ultrafiltration at 14,000 *g* under the same conditions for 30 min to remove salt and other impurities. Finally, the protein solutions were collected and determined by 2-D Quant Kit (GE Healthcare Life Sciences, Pittsburgh, PA, USA) in accordance with the manufacturer’s protocol.

### DIGE Labeling of Striatum Proteins

Each CyDye stock at room temperature was resuspended in anhydrous *N*,*N*-dimethylformamide (DMF), 99.8% (Sigma-Aldrich (Merck) 227056) to achieve a final dye concentration of 1 nmol/μL. The working solutions (200 pmol/μL) of each CyDye used for protein labeling were obtained by diluting the CyDye stock solutions with DMF. After protein quantification, brain protein samples from TRPC1^−/−^ mice and WT mice were diluted to 5 μg/μL. Equal amounts of all samples (25 μg) were mixed to compose a pooled internal standard, and labeled with 200 pmol Cy2 (GE Healthcare, 25–8008–62). The other protein samples (25 μg of each sample) were labeled with 200 pmol Cy3 (GE Healthcare, 25–8008–61) or Cy5 (GE Healthcare, 25–8008–62). All the labeled protein samples were incubated on ice for 30 min. The reaction was quenched subsequently by adding 1 μL of 10 mM lysine (Sigma, L5626) for 10 min. All labeling operations were performed in darkness. Then, the Cy2-, Cy3-, and Cy5-labeled samples were mixed, and 80 μL of 2× lysis buffer (8 M urea, 2% CHAPS, 0.2% DTT, 2% (v/v) IPG buffer, pH 3–11 NL, 0.002% bromophenol blue) was added to each sample. The rehydration buffer was then added to make the total volume of the sample up to 450 μL.

### 2D-DIGE

The first dimension was conducted with the Ettan IPGphor Isoelectric Focusing (IEF) System (GE Healthcare, Pittsburgh, PA, USA). Equal amounts of labeled samples (75 μg) were applied to Immobiline DryStrips (24 cm, pH 3–11 NL) with 2 mL of mineral oil (covered to reduce solvent evaporation). Proteins were taken up onto strips with rehydration at 50 V for 18 h, followed by IEF at 300 V for 12 h, focusing at 500 V for 2 h, step 1000 V for 2 h, gradient 8000 V for 8 h, step 8000 V for 8 h. The room temperature was kept at 18°C. After IEF, each strip was equilibrated with 15 ml of reducing equilibration buffer (6 M urea, 75 mM Tris-HCl buffer (pH 8.8), 30% (v/v) glycerol, 2% (w/v) SDS, and 1% (w/v) DTT) for 15 min. Subsequently, strips were re-equilibrated in another buffer (6 M urea, 75 mM Tris-HCl buffer (pH 8.8), 30% (v/v) glycerol, 2% (w/v) SDS, and 4.5% (w/v) IAA) for 15 min. Every gel was covered with 2 ml of 0.5% (w/v) ultralow melting point agarose sealing solution (25 mM Tris, 192 mM glycine, 0.1% SDS, 0.5% (w/v) agarose, 0.002% (w/v) bromophenol blue). Each equilibrated strip was then loaded on the top of a 12.5% SDS-PAGE gel. Protein separation in the second dimension was run on an Ettan DALTsix Electrophoresis System (GE Healthcare, USA) using the following conditions: 1 W/gel for 1 h, 11 W/gel for 5 h at 18°C in the dark. Following the second dimension, the DIGE gels were immediately scanned using a Typhoon TRIO Variable Mode Imager (GE Healthcare, USA).

### Image Analysis

Gel images were analyzed with the DeCyder software package (Version 6.5, GE Healthcare, USA) following the manufacturer’s protocol. The normalized spot density between the replicate groups was further compared and protein spots found to be statistically significant (*P* < 0.05) identified for analysis.

### In-Gel Digestion

The gels were first stained with Coomassie blue solution (0.12% Coomassie Brilliant Blue G-250, 10% phosphoric acid, 20% ethanol, 10% ammonia sulfate). Spots of interest identified through Decyder software analysis were manually excised from preparative Coomassie blue-stained gel using Eppendorf micropipettes. Gel pieces were destained with 50% acetonitrile (ACN) and 100% ACN, followed by digestion overnight at 37°C with trypsin (Promega Corp., WI, USA) in 15 μL digestion buffer. The tryptic peptides were used for MALDI-TOF-MS/MS analysis.

### Mass Spectrometry

The protein spots were analyzed by MALDI-TOF-MS/MS (AB SCIEX MALDI-TOF/TOF 5800 MS, Foster City, CA, USA). Briefly, a total of 0.6 μL of peptide extract was crystallized with 1 μL 10 mg/mL α-cyano-4-hydroxycinnamic acid (CHCA) in 0.1% TFA, 50% ACN directly on the target and dried at room temperature. The spectra were externally calibrated. Information on mice brain proteins was retrieved from the SwissProt databases (Matrix Science, UK) with MASCOT. The search was performed in the *Mus musculus* database and conducted with a tolerance on mass measurement of 100 ppm in MS mode and 0.3 Da in MS/MS mode. Protein molecular weight (MW) and a fixed carbamidomethyl modification were taken into account when evaluating protein identification.

### Bioinformatics Analysis

Gene ontology (GO) enrichment analysis of the deregulated proteins was performed using DAVID online software following the instructions provided^1^. For the protein-protein interaction prediction analysis, we used STRING database version 10.0, which was embedded in Cytoscape (3.4.0) with a medium confidence threshold 0.4.

### Western-Blot Analysis

Each sample from the above groups was extracted with 400 ml RIPA lysis buffer (Beyotime, Haimen, Jiangsu, China) with protease and phosphatase inhibitor cocktail (Thermo Scientific, Waltham, MA, USA). BCA protein assay kit (Thermo Scientific, USA) was used to determine protein concentration. Equal amounts of proteins were separated by 10% SDS-PAGE gels and then transferred onto PVDF membranes. Membranes containing the transferred proteins were blocked for 1.5 h in 5% skim milk in TBST. Primary antibodies, anti-GRP78 (1:1000, Santa Cruz, sc-376768), anti-protein deglycase (DJ-1) (1:10,000, Abcam, ab76008), anti-dynamin-1 (1:1000, Abcam, ab52611), anti-14–3–3Z (1:1000, Abcam, ab155037), anti-NDUFV2 (1:10,000, Abcam, ab183715), anti-PERK (1:1000, CST, 3192S), anti-p-PERK (1:1000, CST, 3179S), anti-eIF2α (1:1000, Santa Cruz, sc-133132), anti-p-eIF2α (1:1000, CST, 3597S), and anti-CHOP (1:1000, CST, 2895S) were then added and incubated on ice overnight. After washing with TBST, membranes were incubated with anti-rabbit or anti-mouse IgG HRPs (Thermo Fisher Scientific, 1:3000) for 50 min at room temperature. Then the membranes were washed with TBST and treated with enhanced chemiluminescence (ECL) reagents from an ECL kit (Pierce, Thermo Scientific). Blots were detected on a phosphorimager and analyzed according to ImageQuant 1D software (GE Healthcare, USA).

### Statistical Analysis

Data were expressed as the mean ± SEM and analyzed with SPSS 20.0 statistical software (SPSS Inc., Chicago, IL, USA) and GraphPad Prism 7.0 (GraphPad Software, Inc., La Jolla, CA, USA) Statistical analysis among two independent groups was performed by *t*-test. Significant difference of each group was set at *P* < 0.05.

## Results

### The Absence of TRPC1 Caused Neuronal Loss and Apoptosis in Mouse Striatum

**Figure 1:**
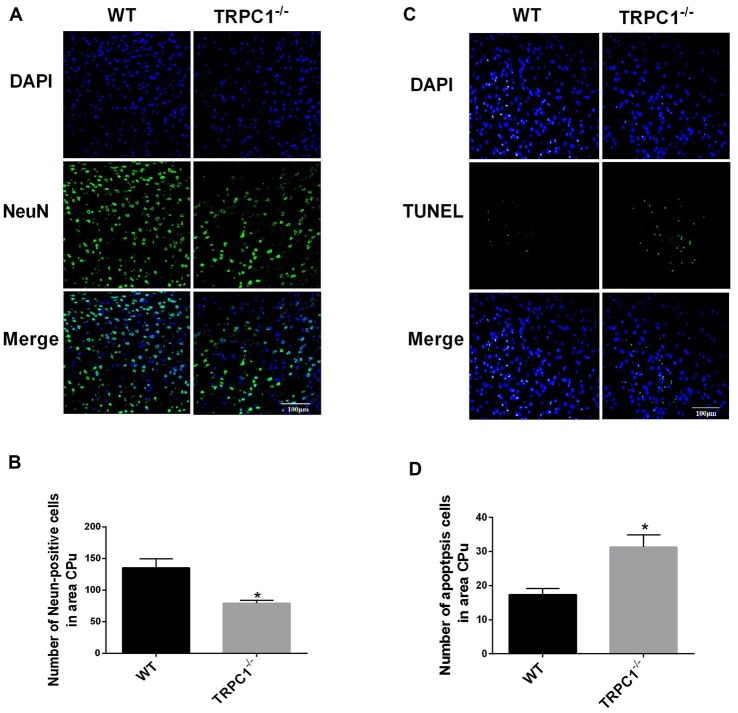
Transient receptor potential channel 1 (TRPC1) deletion caused neuronal loss and apoptosis in striatum. **(A)** Representative immunofluorescent images from the striatum of TRPC1 knockout (TRPC1^−/−^) and wild type (WT) mice; **(B)** The number of NeuN-positive cells in striatum. **(C)** Representative TUNEL staining images from the striatum in TRPC1^−/−^ mice and WT mice. **(D)** The number of apoptotic cells in striatum. The data were expressed as mean ± SEM and statistical analysis between two independent groups was performed by *t*-test. *P < 0.05, vs. WT mice. *n* = 3 for each group. Scale bar = 100 μm.

Significant loss of NeuN-positive cells (a neuron-specific marker) was observed in the striatum of TRPC1^−/−^ mice relative to the WT mice (*P* < 0.01; Figures 1A,B). These data indicate that TRPC1 is required for the survival of neurons in striatum, as previously shown for the hippocampus (*REF*, Xing et al., 2016). TUNEL staining revealed that TUNEL-positive cells were significantly increased in striatum of TRPC1^−/−^ vs. WT mice (*P* < 0.05; Figures 1C,D), indicating that an apoptotic mechanism is involved in the loss of striatal neurons in TRPC1^−/−^ mice.

### Identification of Differentially Expressed Striatal Proteins in TRPC1^−/−^ vs. WT Mice

**Figure 2:**
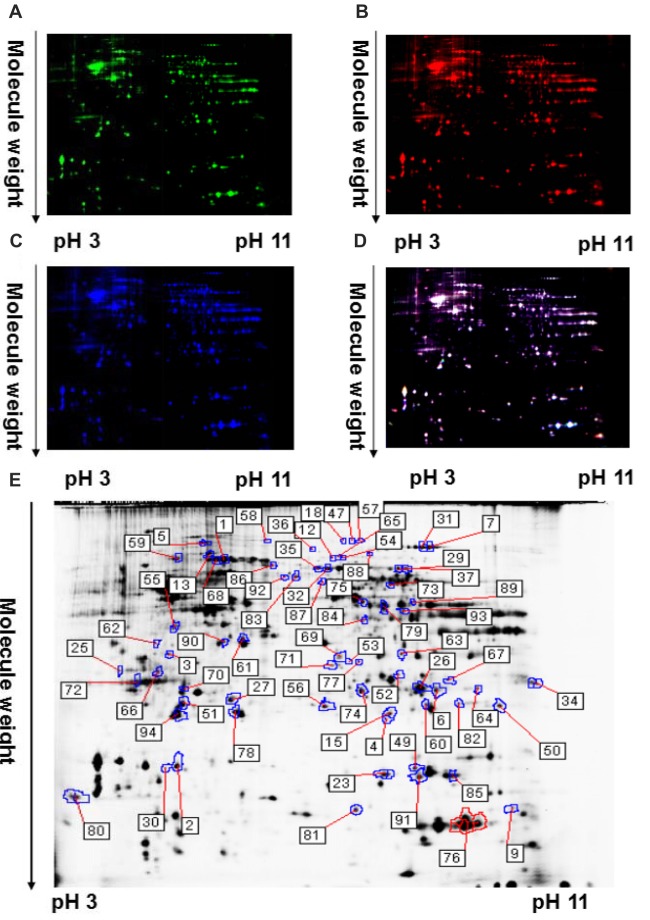
A representative two-dimensional fluorescence difference gel electrophoresis (2D-DIGE) gel image of corpus striatum proteins from TRPC1^−/−^ and WT mice. **(A)** Representative image of striatal proteins from TRPC1^−/−^ mouse brain labeled with Cy5 dye. **(B)** Representative image of a 2D-DIGE gel showing Cy3-labeled striatal proteins from WT mice. **(C)** Representative image of a 2D-DIGE gel showing Cy2-labeled proteins as internal standards. **(D)** A merged image of the 2D-DIGE gel displaying Cy2-, Cy3- and Cy5-labeled proteins. **(E)** Grayscale 2D-DIGE gel image showing 51 differentially expressed protein spots identified by MALDI-TOF-MS/MS (black numbers with white square) in the striatum of TRPC1^−/−^ mice relative to that of WT mice.

**Figure 3:**
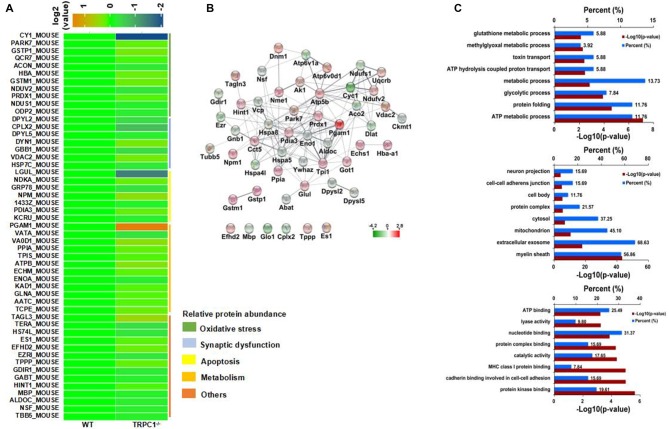
Identification of differentially expressed striatal proteins and bioinformatics analysis in TRPC1^−/−^ vs. WT mice.** (A)** A total of 51 identified differentially expressed striatal proteins included oxidative stress-related proteins, synapse-related proteins, apoptosis-related proteins, metabolism-related proteins and others classified according to their protein functions. **(B)** Protein-protein interaction network among striatal proteins in TRPC1^−/−^ vs. WT mice; proteins are presented as nodes with colors: increased proteins are shown in red while decreased proteins are shown in green. **(C)** Gene ontology (GO) analysis for differentially expressed striatal proteins in TRPC1^−/−^ vs. WT mice.

Protein profile changes in the striatum of TRPC1^−/−^ vs. WT mice were explored with 2D-DIGE and MALDI-TOF-MS/MS. Representative 2D-DIGE gel images of the striatal proteins from TRPC1^−/−^ mice and the WT mice are shown in Figure 2. The spots with a fold-change greater than 1.1 and a *P*-value ≤ 0.05 between TRPC1^−/−^ mice and WT mice were classified as differentially expressed protein spots (*n* = 51) for analysis by MALDI-TOF-MS/MS. Among these 51 protein spots, 26 were increased and 25 were decreased in TRPC1^−/−^ mice compared with that in WT mice. The proteins were listed and classified functionally into oxidative stress-related proteins, synapse-related proteins, apoptosis-related proteins, and metabolism-related proteins according to their established protein functions (Figure 3A).

### Bioinformatics Analysis of Differentially Expressed Proteins

Protein-protein interaction analysis was performed using STRING 10.0 software embedded in Cytoscape 3.5.1. Most of the proteins showed close protein-protein interactions with the other differentially expressed proteins. Only six identified proteins (EFHD2, MBP, GLO1, CPLX2, TPPP and ES1) showed no interaction with the others. Heat shock family proteins HSPA8 and HSPA5, enolase-1 (alpha) (ENO1), phosphoglycerate mutase 1 (PGAM1) and triosephosphate isomerase 1 (TPI1) were shown to be the core proteins in the network (Figure 3B), indicating these proteins could be the key striatal proteins affected by the genetic absence of TRPC1.

All differentially expressed proteins were uploaded to DAVID software for GO analysis. All the enriched items are shown by the percentage and *P*-value and the top 10 enriched items in biological process, cellular components and molecular functions were shown (Figure 3C). Biological processes were most enriched for ATP metabolic process, protein folding and glycolytic process. For the cellular component, myelin sheath, extracellular exosome and mitochondria were the top three enriched items. Protein kinase binding, cadherin binding involved in cell-cell adhesion, and MHC class I protein binding, were the most enriched items among molecular functions.

### TRPC1 Deletion Caused Oxidative Stress in Striatum

**Figure 4:**
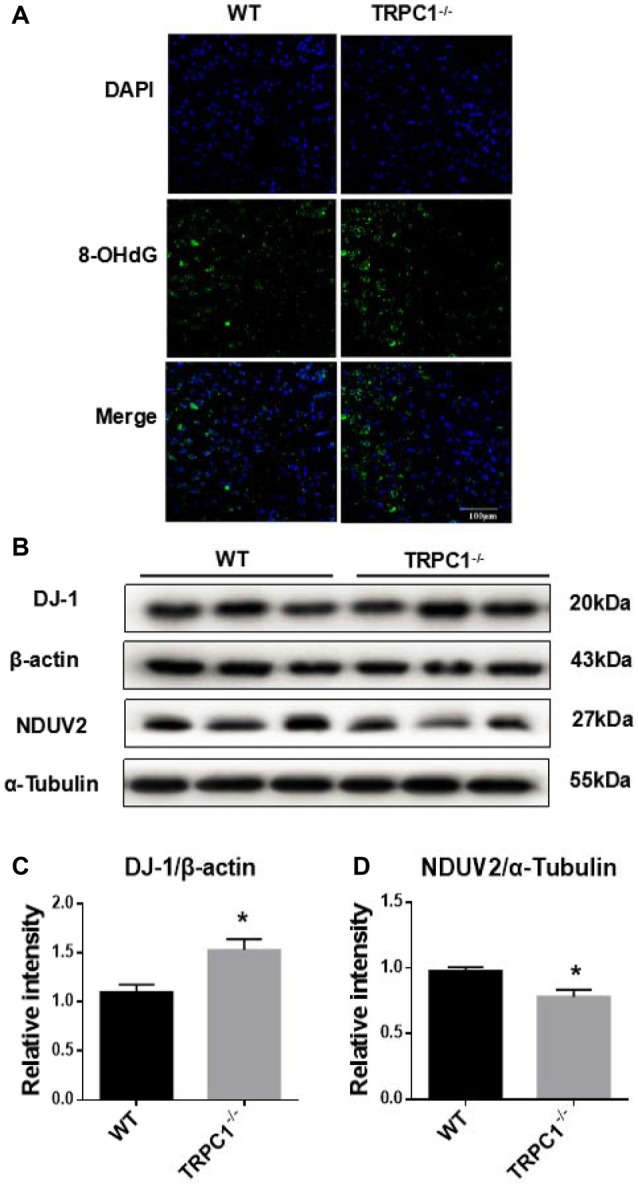
TRPC1 deletion caused DNA oxidative damage in striatum and verification of oxidative stress-related proteins by Western-blot analysis.** (A)** Immunofluorescent images of 8-hydroxy-2′-deoxyguanosine (8-OHdG) staining of TRPC1^−/−^ and WT mice striatum. **(B,C)** Relative intensity of protein deglycase (DJ-1) in striatum. **(B,D)** Relative intensity of NADH dehydrogenase (ubiquinone) flavoprotein 2 (NDUV2) in striatum. Data expressed as mean ± SEM. Statistical analysis between two independent groups was performed by *t*-test. *P < 0.05, vs. WT mice. *n* = 3 per group. Scale bar = 100 μm.

In the proteomic study, we found differentially expressed proteins were closely related with mitochondria and ATP metabolism, suggesting that oxidative stress may be involved in the increased apoptosis the striatum of mice lacking TRPC1. To reveal the potential influence of TRPC1 on oxidative stress in striatum, we performed an immunofluorescence staining of striatum using an antibody to 8-hydroxy-2′-deoxyguanosine (8-OHdG), a stable and integral marker of DNA oxidative damage. Oxidative damage in cellular DNA was significantly increased in striatum of TRPC1^−/−^ mice compared to that of WT mice (*P* < 0.05; Figure 4A). Furthermore, by Western-blot analysis, two proteins, mitochondrial NADH dehydrogenase (ubiquinone) flavoprotein 2 (NDUV2) and DJ-1 (also known as Parkinson disease protein 7), of a total of 11 oxidative stress-related proteins identified by proteomic analysis, were validated respectively to be decreased and increased in the striatum of TRPC1^−/−^ mice compared with that of the WT mice (Figures 4B–D).

### Validation of Apoptosis-Related Proteins

**Figure 5:**
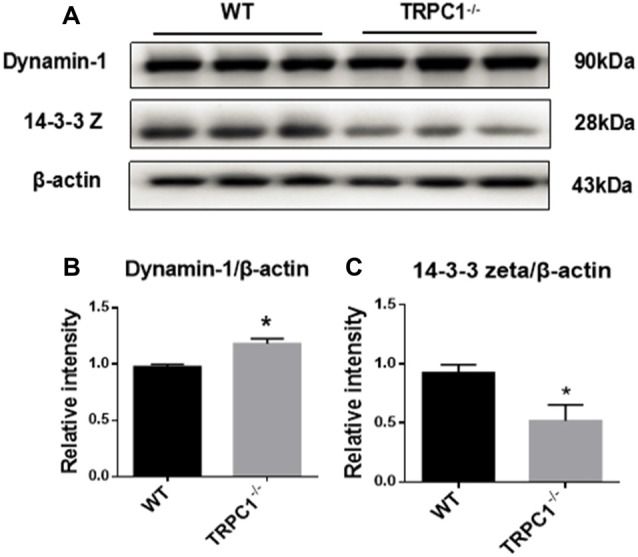
Validation of differentially expressed apoptosis-related proteins by Western-blot analysis.** (A,B)** Relative intensity of dynamin-1. **(A,C)** Relative intensity of 14–3–3Z protein. Data expressed as mean ± SEM and statistical analysis between two independent groups was performed by *t*-test. *P < 0.05, vs. WT mice. *n* = 3 for each group.

Western-blot analysis was used to further validate the expression of some apoptosis-related proteins identified by proteomic analysis. Apoptosis-related protein 14-3-3 Z was significantly decreased while dynamin-1 (D2 dopamine (DA) receptor binding, apoptosis-related protein) was increased in the striatum of TRPC1^−/−^ mice relative to WT mice (Figure 5).

### ER Stress Was Induced in Striatum by TRPC1 Deletion

**Figure 6:**
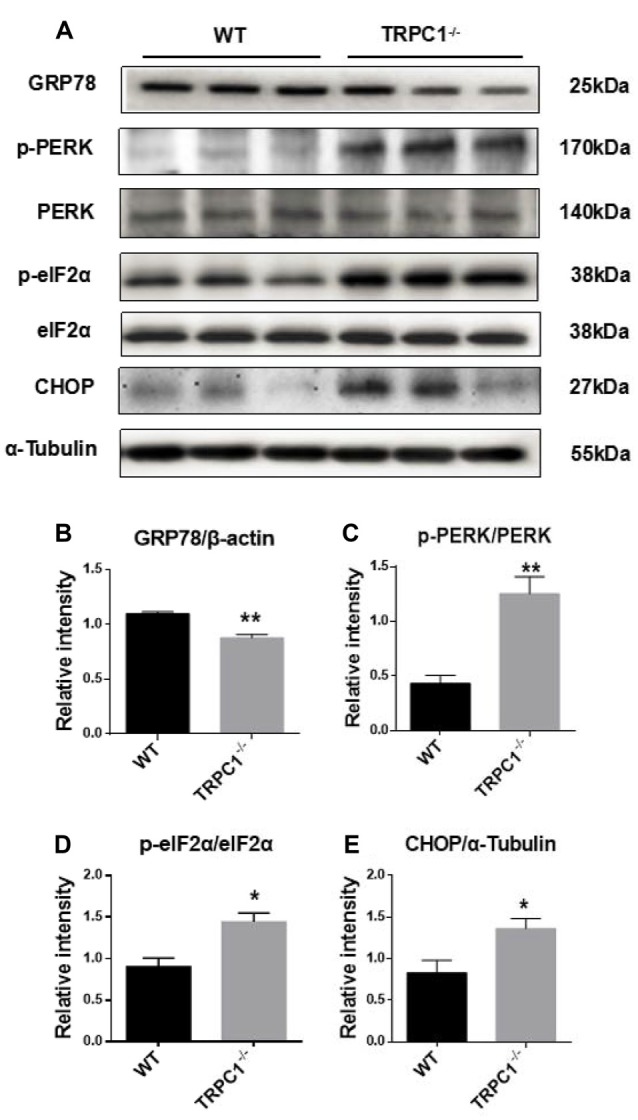
Validation of differentially expressed endoplasmic reticulum (ER) stress-related proteins in striatum by Western-blot analysis.** (A,B)** The relative intensity of GRP78. **(A,C)** The relative intensity of p-PERK/PERK. **(A,D)** The relative intensity of p-eIF2α/eIF2α. **(A,E)** The relative intensity of CHOP. Data expressed as mean ± SEM and statistical analysis between two independent groups was performed by *t*-test. **P < 0.01 and *P < 0.05, vs. the WT mice. *n* = 3 for each group.

Since proteomic analysis showed that endoplasmic reticulum (ER) stress-related proteins HSPA8 and HSPA5 were induced in the striatum by TRPC1 deletion (see above), we asked whether ER stress-related signaling pathways were also activated. Western-blot analysis showed significant decrease of GRP78, a key chaperone protein involved in ER stress in the striatum of TRPC1^−/−^ mice. Furthermore, we found that the ER stress signaling molecules p-PERK, p-eIF2α and CHOP were significantly increased in striatum of TRPC1^−/−^ mice (Figure 6). These data indicated that TRPC1 deletion triggered ER stress in the mouse striatum.

## Discussion

TRPC1 is consistently associated with store-operated calcium (Ca^2+^) entry (SOCE), an important regulatory mechanism for Ca^2+^ homeostasis, which is closely related to neuronal development and differentiation (Inglefield et al., 2001; Wu et al., 2004). Ca^2+^ signaling is of vital importance for multiple physiological processes, including neuronal development/differentiation/maturation, neurosecretion and exocytosis (Bollimuntha et al., 2017; Chen et al., 2017).

TRPC1 is a subunit of heteromeric channel complexes that function in the regulation of Ca^2+^ permeability (Storch et al., 2012). TRPC1 and the rest of its family members are nonselective cation-permeable channels (Gees et al., 2010). Activation of TRPC is linked with the activation of plasma membrane-associated GPCRs or RTKs, which induce the hydrolysis of phosphatidylinositol 4,5-bisphosphate (PIP2) into IP3, and finally cause the deletion of ER Ca^2+^ stores (Bollimuntha et al., 2017). Neurotoxin-induced ER stress is also regulated by TRPC1 mediated AKT/mTOR signaling in dopaminergic neurons (Selvaraj et al., 2012). TRPC1 is also involved in the suppression of Cav_1.3_ activity through the stromal interacting molecule-1 (STIM1) to protect the dopaminergic neurons (Sun et al., 2017). However, the potential mechanism underlying TRPC1-mediated neuronal loss/apoptosis, such as the striatal neuronal apoptosis we observed here, is not clear.

High-throughput proteomic analysis is an efficient approach to gain insight into pathophysiological mechanisms of dysfunction and diseases. In this study, using 2D-DIGE coupled with MS, we revealed a total of 51 differentially expressed proteins (26 increased and 25 decreased) in the striatum of TRPC1^−/−^ mice compared with WT mice. Bioinformatics analysis revealed that these dysregulated proteins were associated with ER stress, glycolytic process, oxidative stress and apoptosis.

### ER Stress and Apoptosis

Protein synthesis and folding is thought to be a pivotal ER function. Various conditions can lead to ER stress (Mori, 2000) which engages the unfolded protein response (UPR), an adaptive signal transduction pathway (Tabas and Ron, 2011; Hetz et al., 2015). Three UPR sensors activating transcription factor 6 (ATF6α), protein kinase RNA-like ER kinase (PERK) and endoribonuclease inositol-requiring enzyme 1-alpha (IRE1α) are important for the protein folding process through ER (Tirasophon et al., 1998; Harding et al., 1999; Haze et al., 1999). All three sensors are maintained in an inactive state with the ER chaperone, GRP78. Upon accumulation of unfolded proteins, three sensors are released and activate, triggering the UPR, a pro-survival response that can restore ER function. However, if protein aggregation is persistent, signaling will switch from pro-survival to pro-apoptosis. TRPC1 is involved in the regulation of Ca^2+^ homeostasis and the inhibition of UPR, which contribute to the survival of neurons (Selvaraj et al., 2012). Release of PERK from GRP78 leads to kinase dimerization and autophosphorylation (Szegezdi et al., 2006). Activated PERK phosphorylates eukaryotic initiation factor 2 (eIF2) and leads to the induction of CHOP, also called GADD153 or DDIT-3 as transcription factor, which is important for the regulation of apoptosis and cell death pathway. The downregulation of GRP78 by small interfering RNAs resulted in exacerbating neurotoxicity of alpha-synuclein (α-syn) in nigral DA neurons (Salganik et al., 2015) Silencing of TRPC1 or STIM1 can raise neurotoxin-induced loss of SOCE, the associated increase in ER Ca^2+^ levels and the resultant UPR (Selvaraj et al., 2012). Taken together, we speculated that striatal neuronal loss caused by TRPC1 deletion is possibly related to an increase in ER Ca^2+^ levels and the resultant UPR through down-regulation of GRP78. Furthermore, we found that phosphorylation of PERK and eIF2α was increased in the absence of TRPC1 in striatum and the increase of CHOP through Western-blot analysis. These results indicated that the p-PERK/p-eIF2α mediated CHOP pathway participates in the ER stress event in the absence of TRPC1.

### Oxidative Stress and Apoptosis

Oxidative stress, caused by oxidative disturbance, can induce apoptosis via both mitochondria-dependent and independent pathways (Sinha et al., 2013). In the proteomic studies, we found that various oxidative stress-related and apoptosis-related proteins were dysregulated in the absence of TRPC1. Protein DJ-1, a multi-function factor for transcription and molecular chaperone, is upregulated in oxidative stress or active astrocytes (Kahle et al., 2009; Yanagida et al., 2009). There are also studies demonstrating that the upregulation of DJ-1 protects neurons from oxidative stress (Zhou and Freed, 2005; Billia et al., 2013). We found that DJ-1 was increased in the striatum of TRPC1^−/−^ mice, which suggests that a mitochondria-independent antioxidant pathway is activated. NDUV2 is one of the core subunits of mitochondrial Complex I, gene mutation of which causes functional loss of NDUV2, which is linked with various diseases, including neurodegenerative disease (Mimaki et al., 2012). We found NDUV2 was decreased in striatum of the TRPC1^−/−^ mouse. These results suggest that both the mitochondria-dependent and -independent pathways may be involved in the oxidative stress pathway. Furthermore, the staining of 8-OHdG, a stable and integral marker of DNA oxidative damage, intuitively reflects the influence of TRPC1 KO on oxidative stress in striatum. TRPC1 stimulated neuronal stem cell proliferation in the manner of basic fibroblast growth factor/fibroblast growth factor receptor-1 ((bFGF/FGFR-1)-induced Ca^2+^ influx (Fiorio Pla et al., 2005). Our data indicate that the increased reactive oxygen species (ROS) production, Ca^2+^ entry and glutathione (GSH) depletion, may lead to apoptotic cell death in the absence of TRPC1. Additionally, in the bioinformatics analysis, the differentially expressed proteins in TRPC1^−/−^ vs. WT were mostly enriched in ATP metabolic process, thereby revealing a link between TRPC1 and oxidative stress. Thus, TRPC1 plays a role in the induction of oxidative stress and apoptosis in striatal cells.

### Apoptosis-Related Signaling Molecules and Apoptosis

The neuron-specific protein dynamin-1 is a multidomain GTPase that plays a pivotal role in the fission stage of synaptic vesicle recycling and vesicle trafficking. DYN1 is also required for the maturation of apoptotic cells engulfed into endosomes (Kinchen et al., 2008). DYN1 can translocate Fas protein from the Golgi apparatus to the cell surface and enable it to bind with its ligand (FasL), and then induce extrinsic apoptosis and caspase-dependent cell death (Ivanov et al., 2006). The overexpression of 70-kDa inducible Hsp (Hsp70) attenuated Fas-mediated apoptotic death and induced downregulation of dynamin-1 (Kim et al., 2016). In our study, we also found significant striatal neuronal apoptosis and increase of dynamin-1 with TRPC1 deletion, indicating that dynamin-1 is involved in apoptosis associated with deletion of the gene encoding TRPC1.

## Conclusion

**Figure 7:**
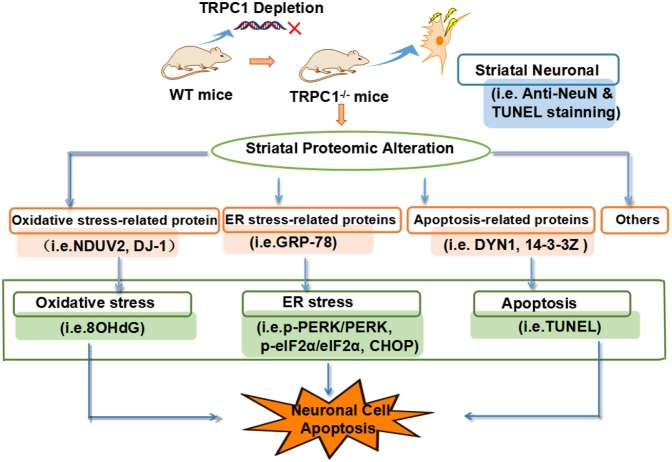
TRPC1 deletion-induced caused striatal neuronal apoptosis is attributed to the dysregulation of proteins related to the biological/pathogenic processes, ER stress, oxidative stress and apoptosis-related signaling.

We showed that deletion of the gene encoding TRPC1 causes striatal neuronal loss and apoptosis associated with significant changes in protein expression. These dysregulated proteins were mainly involved in biological/pathological processes relating to ER stress, oxidative stress and apoptosis. Taken together, we conclude that TRPC1 deletion causes striatal neuronal loss/apoptosis by disturbing multiple biological processes, including ER function, oxidative stress, and apoptosis-related signaling (Figure 7). These data suggest that TRPC1 is a key player in the regulation of striatal cellular survival and death.

## Author Contributions

DW and HY performed the experiments, analyzed the data and drafted the manuscript. ZZ, YG, XR, BX, JY, JL, XY and HX designed the study and analyzed the data. PSS, XY and HX wrote and revised the manuscript.

## Conflict of Interest Statement

The authors declare that the research was conducted in the absence of any commercial or financial relationships that could be construed as a potential conflict of interest.

## Footnotes

^1^https://david.ncifcrf.gov/

## References

[B1] Arshad A. Chen X. Cong Z. Qing H. Deng Y. (2014). TRPC1 protects dopaminergic SH-SY5Y cells from MPP^+^, salsolinol, and *N*-methyl-(*R*)-salsolinol-induced cytotoxicity. Acta Biochim. Biophys. Sin. 46, 22–30. 10.1093/abbs/gmt127 24252728

[B2] Billia F. Hauck L. Grothe D. Konecny F. Rao V. Kim R. H.. (2013). Parkinson-susceptibility gene DJ-1/PARK7 protects the murine heart from oxidative damage *in vivo*. Proc. Natl. Acad. Sci. U S A 110, 6085–6090. 10.1073/pnas.1303444110 23530187 PMC3625258

[B3] Bollimuntha S. Pani B. Singh B. B. (2017). Neurological and motor disorders: neuronal store-operated Ca^2+^ signaling: an overview and its function. Adv. Exp. Med. Biol. 993, 535–556. 10.1007/978-3-319-57732-6_27 28900932 PMC5821072

[B4] Chen C. Ma Q. Deng P. Yang J. Yang L. Lin M.. (2017). Critical role of TRPC1 in thyroid hormone-dependent dopaminergic neuron development. Biochim. Biophys. Acta 1864, 1900–1912. 10.1016/j.bbamcr.2017.07.019 28779972

[B5] Damann N. Voets T. Nilius B. (2008). TRPs in our senses. Curr. Biol. 18, R880–R889. 10.1016/j.cub.2008.07.063 18812089

[B6] Fiorio Pla A. Maric D. Brazer S. C. Giacobini P. Liu X. Chang Y. H.. (2005). Canonical transient receptor potential 1 plays a role in basic fibroblast growth factor (bFGF)/FGF receptor-1-induced Ca^2+^ entry and embryonic rat neural stem cell proliferation. J. Neurosci. 25, 2687–2701. 10.1523/jneurosci.0951-04.2005 15758179 PMC6725156

[B7] Gees M. Colsoul B. Nilius B. (2010). The role of transient receptor potential cation channels in Ca^2+^ signaling. Cold Spring Harb. Perspect. Biol. 2:a003962. 10.1101/cshperspect.a003962 20861159 PMC2944357

[B9] Harding H. P. Zhang Y. Ron D. (1999). Protein translation and folding are coupled by an endoplasmic-reticulum-resident kinase. Nature 397, 271–274. 10.1038/16729 9930704

[B10] Haze K. Yoshida H. Yanagi H. Yura T. Mori K. (1999). Mammalian transcription factor ATF6 is synthesized as a transmembrane protein and activated by proteolysis in response to endoplasmic reticulum stress. Mol. Biol. Cell 10, 3787–3799. 10.1091/mbc.10.11.3787 10564271 PMC25679

[B11] Hetz C. Chevet E. Oakes S. A. (2015). Proteostasis control by the unfolded protein response. Nat. Cell Biol. 17, 829–838. 10.1038/ncb3184 26123108 PMC5546321

[B12] Hong C. Seo H. Kwak M. Jeon J. Jang J. Jeong E. M.. (2015). Increased TRPC5 glutathionylation contributes to striatal neuron loss in Huntington’s disease. Brain 138, 3030–3047. 10.1093/brain/awv188 26133660 PMC4643628

[B13] Inglefield J. R. Mundy W. R. Shafer T. J. (2001). Inositol 1,4,5-Triphosphate receptor-sensitive Ca^2+^ release, store-operated Ca^2+^ entry, and cAMP responsive element binding protein phosphorylation in developing cortical cells following exposure to polychlorinated biphenyls. J. Pharmacol. Exp. Ther. 297, 762–773. 11303068

[B14] Ivanov V. N. Ronai Z. Hei T. K. (2006). Opposite roles of FAP-1 and dynamin in the regulation of Fas (CD95) translocation to the cell surface and susceptibility to Fas ligand-mediated apoptosis. J. Biol. Chem. 281, 1840–1852. 10.1074/jbc.m509866200 16306044 PMC4376329

[B15] Kahle P. J. Waak J. Gasser T. (2009). DJ-1 and prevention of oxidative stress in Parkinson’s disease and other age-related disorders. Free Radic. Biol. Med. 47, 1354–1361. 10.1016/j.freeradbiomed.2009.08.003 19686841

[B16] Kim J. Y. Kim N. Zheng Z. Lee J. E. Yenari M. A. (2016). 70-kDa heat shock protein downregulates dynamin in experimental stroke: a new therapeutic target? Stroke 47, 2103–2111. 10.1161/STROKEAHA.116.012763 27387989 PMC4961549

[B17] Kinchen J. M. Doukoumetzidis K. Almendinger J. Stergiou L. Tosello-Trampont A. Sifri C. D.. (2008). A novel pathway for phagosome maturation during engulfment of apoptotic cells. Nat. Cell Biol. 10, 556–566. 10.1038/ncb1718 18425118 PMC2851549

[B18] Mimaki M. Wang X. McKenzie M. Thorburn D. R. Ryan M. T. (2012). Understanding mitochondrial complex I assembly in health and disease. Biochim. Biophys. Acta 1817, 851–862. 10.1016/j.bbabio.2011.08.010 21924235

[B19] Montell C. (2005). The TRP superfamily of cation channels. Sci. STKE 2005:re3. 10.1126/stke.2722005re3 15728426

[B20] Mori K. (2000). Tripartite management of unfolded proteins in the endoplasmic reticulum. Cell 101, 451–454. 10.1016/s0092-8674(00)80855-7 10850487

[B21] Salganik M. Sergeyev V. G. Shinde V. Meyers C. A. Gorbatyuk M. S. Lin J. H.. (2015). The loss of glucose-regulated protein 78 (GRP78) during normal aging or from siRNA knockdown augments human α-synuclein (α-syn) toxicity to rat nigral neurons. Neurobiol. Aging 36, 2213–2223. 10.1016/j.neurobiolaging.2015.02.018 25863526 PMC4433578

[B22] Selvaraj S. Sun Y. Watt J. A. Wang S. Lei S. Birnbaumer L.. (2012). Neurotoxin-induced ER stress in mouse dopaminergic neurons involves downregulation of TRPC1 and inhibition of AKT/mTOR signaling. J. Clin. Invest. 122, 1354–1367. 10.1172/JCI61332 22446186 PMC3314472

[B23] Selvaraj S. Watt J. A. Singh B. B. (2009). TRPC1 inhibits apoptotic cell degeneration induced by dopaminergic neurotoxin MPTP/MPP^+^. Cell Calcium 46, 209–218. 10.1016/j.ceca.2009.07.008 19695701 PMC2856605

[B24] Sinha K. Das J. Pal P. B. Sil P. C. (2013). Oxidative stress: the mitochondria-dependent and mitochondria-independent pathways of apoptosis. Arch. Toxicol. 87, 1157–1180. 10.1007/s00204-013-1034-4 23543009

[B25] Storch U. Forst A.-L. Philipp M. Gudermann T. Mederos y Schnitzler M. (2012). Transient receptor potential channel 1 (TRPC1) reduces calcium permeability in heteromeric channel complexes. J. Biol. Chem. 287, 3530–3540. 10.1074/jbc.M111.283218 22157757 PMC3271006

[B26] Strübing C. Krapivinsky G. Krapivinsky L. Clapham D. E. (2003). Formation of novel TRPC channels by complex subunit interactions in embryonic brain. J. Biol. Chem. 278, 39014–39019. 10.1074/jbc.M306705200 12857742

[B27] Sun Y. Zhang H. Selvaraj S. Sukumaran P. Lei S. Birnbaumer L.. (2017). Inhibition of L-type Ca^2+^ channels by TRPC1-STIM1 complex is essential for the protection of dopaminergic neurons. J. Neurosci. 37, 3364–3377. 10.1523/JNEUROSCI.3010-16.2017 28258168 PMC5373123

[B28] Szegezdi E. Logue S. E. Gorman A. M. Samali A. (2006). Mediators of endoplasmic reticulum stress-induced apoptosis. EMBO Rep. 7, 880–885. 10.1038/sj.embor.7400779 16953201 PMC1559676

[B29] Tabas I. Ron D. (2011). Integrating the mechanisms of apoptosis induced by endoplasmic reticulum stress. Nat. Cell Biol. 13, 184–190. 10.1038/ncb0311-184 21364565 PMC3107571

[B30] Tirasophon W. Welihinda A. A. Kaufman R. J. (1998). A stress response pathway from the endoplasmic reticulum to the nucleus requires a novel bifunctional protein kinase/endoribonuclease (Ire1p) in mammalian cells. Genes Dev. 12, 1812–1824. 10.1101/gad.12.12.1812 9637683 PMC316900

[B31] Venkatachalam K. Montell C. (2007). TRP channels. Annu. Rev. Biochem. 76, 387–417. 10.1146/annurev.biochem.75.103004.142819 17579562 PMC4196875

[B32] Wang Z. Wang Y. Tian X. Shen H. Dou Y. Li H.. (2016). Transient receptor potential channel 1/4 reduces subarachnoid hemorrhage-induced early brain injury in rats via calcineurin-mediated NMDAR and NFAT dephosphorylation. Sci. Rep. 6:33577. 10.1038/srep33577 27641617 PMC5027540

[B33] Wu X. Zagranichnaya T. K. Gurda G. T. Eves E. M. Villereal M. L. (2004). A TRPC1/TRPC3-mediated increase in store-operated calcium entry is required for differentiation of H19–7 hippocampal neuronal cells. J. Biol. Chem. 279, 43392–43402. 10.1074/jbc.M408959200 15297455

[B34] Xing R. Zhang Y. Xu H. Luo X. Chang R. C.-C. Liu J.. (2016). Spatial memory impairment by TRPC1 depletion is ameliorated by environmental enrichment. Oncotarget 7, 27855–27873. 10.18632/oncotarget.8428 27034165 PMC5053693

[B35] Yanagida T. Tsushima J. Kitamura Y. Yanagisawa D. Takata K. Shibaike T.. (2009). Oxidative stress induction of DJ-1 protein in reactive astrocytes scavenges free radicals and reduces cell injury. Oxid. Med. Cell. Longev. 2, 36–42. 10.4161/oxim.2.1.7985 20046643 PMC2763229

[B36] Zeng C. Tian F. Xiao B. (2016). TRPC channels: prominent candidates of underlying mechanism in neuropsychiatric diseases. Mol. Neurobiol. 53, 631–647. 10.1007/s12035-014-9004-2 25502458

[B37] Zhou W. Freed C. R. (2005). DJ-1 up-regulates glutathione synthesis during oxidative stress and inhibits A53T α-synuclein toxicity. J. Biol. Chem. 280, 43150–43158. 10.1074/jbc.M507124200 16227205

